# Beatrice Hinkle and the Early History of Jungian Psychology in New York

**DOI:** 10.3390/bs3030492

**Published:** 2013-08-20

**Authors:** Jay Sherry

**Affiliations:** 34 Plaza Street E., #1109, Brooklyn, NY 11238, USA; E-Mail: jay.sherry@verizon.net

**Keywords:** Beatrice Hinkle, Heterodoxy Club, Liberal Club, feminism, Provincetown Players, progressive education, The Analytical Psychology Club of New York

## Abstract

As the leading proponent of psychoanalysis, Jung made trips to New York in 1912 and 1913. The first was to give his Fordham lectures, the second has escaped notice but was crucial in the early dissemination of Jungian psychology in the U.S. This paper will elaborate on this development by highlighting the career and influence of Beatrice Hinkle, the country’s first Jungian psychoanalyst. She was an M.D. and ardent feminist who introduced Jung to her Greenwich Village circle, translated his magnum opus *Transformations and Symbols of the Libido*, and helped establish the institutional basis of Jungian psychology in America.

As the gaps in the history of Jungian psychology in the United States continue to be filled in, its place in modern American cultural life will need reassessing. The work of Burnham, Hale, and Taylor make clear Jung’s contributions to American psychiatry and psychotherapy but, after his break with Freud, he was to be increasingly marginalized in the professional literature [1,2,3]. Charles Oberndorf, an early New York analyst, explained it this way in his history of psychoanalysis in America. “Jung’s later works contain so much of spiritual, mystical, and quasi-theological admixtures, which he engrafted on Freud’s basic theories in the attempt to defend ‘religious instincts,’ that it is questionable whether his concepts would still come within any broad definition of psychoanalysis. His theory and procedures have appealed strongly to the inexactitude and fantasy of many laymen” ([4], pp. 132–133). This paper will discuss the early history of Jungian psychology in New York, its first outpost in the United States, using the life and career of Beatrice Hinkle, the country’s first Jungian psychoanalyst, as its lens.

She is now best-remembered as the translator of Jung’s *Wandlungen und Symbole der Libido* as *Psychology of the Unconscious*, a book now known as *Symbols of Transformation* [5]. Her translation was one of the publishing sensations of 1916 and she single-handedly kept it in print for the next thirty years. She was one of the many Progressive-era women with college degrees who moved to New York to stake out their professional careers. Research by Judith Schwarz and Kate Wittenstein in the 1980s documented Hinkle’s membership in the Heterodoxy Club, America’s first feminist organization [6,7]. This fact means that her career must now be contextualized as that of a pioneering feminist who championed Jung’s new approach to the creative potential of the psyche as a powerful tool for personal and social transformation. 

One unfortunate fact for researchers is that her personal papers were destroyed after her death as stipulated in her will [8]. This is a great loss to Jungian historiography as they would have included letters from Jung, spanning the entire development of his thought. The Kristine Mann Library in New York does have a Hinkle file, but a note therein indicates that, at some point, many of its contents were disposed of. Her family has a collection of more personal documents from various stages of her life.

Although 1874 is routinely given as the year of Hinkle’s birth, horoscopes in their possession make certain that the date was actually 1870 since in one “1870” is crossed out and over-written with “1874” and on the other the year was ripped off [9]. She was born on October 10^th^ to B. Frederick and Elizabeth Moses and raised in San Francisco. The daughter of one lawyer she married another, Walter Scott Hinkle, an assistant district attorney. After having a son, Walter, and a daughter, Consuelo, she decided to become a doctor and in 1899 graduated from Cooper Medical College, which later became part of Stanford University [10]. She was appointed San Francisco’s city physician and so became the first woman in the U.S. to hold that position. She developed an interest in the emotional dimension of public health issues after noticing that during a bubonic plague her rate of success was higher than that of other doctors. She later said that the reason for this was that she “poured her own vitality, her own belief into the patient” and that a doctor’s work was to “reinforce the spirit that makes for recovery” ([11], pp. 46–47). After the death of her husband she moved to New York City in 1905 where she took up residence at #10 Gramercy Park where the influential realist painter and teacher Robert Henri had his studio; she later owned a brownstone down the block at #31. One colleague remembered that “she looked into all the various kinds of cures, or isms, like New Thought, Christian Science and hypnotism, (*etc*.) ... [and that] Her energy was always superlative and her undivided interest in anything she was doing was outstanding” [12]. After spending time upstate on the staff of a New Thought sanatorium in Kingston, NY, she joined the staff headed by Charles L. Dana, America’s leading neurologist, at Cornell Medical College where they opened one of the first psychotherapy clinics in the U.S. [13]. In 1909 she wrote that “several German physicians have devised methods for bringing up from the depths of the patients’ mind circumstances and incidents forgotten by them at the present time…” ([14], pp. 16–17).

Around this time she married Philip Garrett Eastwick, a businessman, and went to Europe to study psychoanalysis. She attended the 1911 Psychoanalytic Congress, traveling to Weimar by train in the company of Freud and Jung [15]. After her return from Europe Hinkle rejoined the Cornell staff and began a private analytical practice. She expressed her new method in the following quote “It is this definite recognition and use of the emotions and feelings in the service of the patient himself which constitutes one of the many differences between the so called persuasion and advice giving reeducational methods, and psychoanalysis. In the latter method no advice or persuasion, nor suggestion of any sort is given. Everything must come from the patient and be felt and understood by him for himself” ([16], p. 1085). She would likely have attended some or all of Jung’s 1912 Fordham lectures that were soon published as “The theory of psychoanalysis” in the inaugural issue of *Psychoanalytic Review*, the first American journal devoted to the new field. She arranged for Charlotte Teller, one of her protégés, to interview Jung for the New York Times when he was in town for the conference. Teller (1876–1953) was an aspiring writer who had lived at the A Club, a collective apartment house run by a group of Greenwich Village radicals. She wrote to a friend “I met Jung on Wednesday the day he arrived at Dr. Hinkle’s. He has a quick sense of humor and good English at his command. We walked up Fifth Avenue afterwards and he spoke of a prophetic dream about me…” [17].

Hinkle was a member of the Liberal Club, one of New York’s leading reform clubs, which was located at 132 19th Street, only a block from her Gramercy Park home. It was an intellectual forum for discussing such issues as birth control, divorce, and the labor struggle and counted among its other members the muckraking journalist Lincoln Steffens. It turns out that the two were also fellow members of the California Club on whose reception committee Hinkle served in 1911. Another member was Percy Stickney Grant, the rector of the Episcopal Church of the Ascension on Fifth Avenue, who had begun the Public Forum at his church where current social issues were debated on Sunday evenings. In March, 1913, Jung spoke to the Club about “Dreams” and although no record of the talk seems to exist, we can get some idea of what he said from a paper he wrote shortly afterward. In it he wrote that “anyone keenly interested in the dream problem cannot have failed to observe that a dream has also a *progressive* continuity ... since dreams occasionally exert a remarkable influence upon the conscious mental life... These occasional after-effects are usually seen in a more or less distinct change in the dreamer’s frame of mind” ([18], p. 299).

Later that year a radical high school teacher, Henrietta Rodman, engineered a split with the more mainstream members of the Club and moved it down to MacDougal Street where it became the unofficial headquarters for the rebellious young people gravitating to Greenwich Village. Many of them worked in settlement houses where they helped newly-arrived immigrants adjust to their new urban environment. Most of them found socialism to be the most promising solution to the inequities and injustices created by industrial capitalism and voted for Eugene Debs in the election of 1912 that was won by the Democrat Woodrow Wilson. 

Jung’s celebrity status in New York coincided with that enjoyed by the French philosopher Henri Bergson who also visited the city in 1913. Hinkle noted this connection when she wrote that “It is most interesting, therefore, to find that a modern philosopher, Henri Bergson, has evolved a philosophy by an entirely different route, corroborative and analogous, in many of its conceptions, with that which analytical psychology has produced . . . [psychic energy] is called by Dr. Jung, the *libido*, and will be recognized as similar to Bergson’s *élan vital* or ‘creative energy’” ([16], p. 1081).

Hinkle introduced Jung to her down-town avant-garde circle. She took him to a dinner party at Patchin Place hosted by some members of the Heterodoxy Club. It had been founded in 1912 by a group of feminists who met every other Saturday until the 1940s. One of their husbands, Carl Zigrosser, later related an anecdote from that evening. “Guests ranged from university professors and writers to distinguished labor administrators... Patchin still talked about a visit by the famous analyst, Carl G. Jung. The atmosphere had been rather stiff and formal until Jung broke the ice by addressing a pet dog who was misbehaving with his leg: ‘Come, come, be reasonable, I’m not a female’” ([19], pp. 100–101). One of the Heterodites, Mary Alden Hopkins, soon left for Zurich where she did analysis with Maria Moltzer, Jung’s research assistant. Hinkle introduced Jung to Kahlil Gibran who drew his pencil portrait, most likely at his studio apartment nearby. It is also probable that she accompanied Jung to the famous Armory Show of modern art, which was being held at the National Guard armory just a few blocks north of her Gramercy Park apartment. Her neighbor, Robert Henri, was involved in the show and one wonders if Hinkle introduced the two men. 

Blackballed by the New York Psychoanalytic Society in 1915 for her allegiance to Jung, Hinkle still remained one of New York’s leading psychoanalysts along with A. A. Brill and Smith Ely Jelliffe; she was to remain in contact with several members of the more eclectic American Psychoanalytic Association such as Trigant Burrow and L. Pierce Clark. She encouraged a wealthy patient, Annette Rankine, to subsidize the *Seven Arts*, a literary journal being started by James Oppenheim another of her patients. The story goes that she encouraged Rankine to do something constructive with her wealth who then sold her collection of Whistlers to raise the necessary capital. After publishing such up-and-coming literary figures as Robert Frost, Eugene O’Neill, and D.H. Lawrence it was forced to close because of Rankine’s disapproval of its outspoken opposition to America’s entry into World War I.

Hinkle’s translation of Jung was widely reviewed and became one of the publishing sensations of 1916. Eugene O’Neill said that “The book that interested me the most of the Freudian school is Jung’s *Psychology of the Unconscious*… If I have been influenced unconsciously, it must have been by this book more than any other...” ([20], p. 245). Jack London said that after reading it he was “standing on the edge of a world so new, so terrible, so wonderful that I am almost afraid to look over into it” ([21], p. 323). Encouraged by reading Jung to drink from the well of world mythology London wrote a series of tales narrated by a Polynesian story-teller that was published after his death as *Tales from a Makaloa Mat* (1919). He animated them with a philosophy decidedly more humane and optimistic than the Social Darwinism found in such earlier works as *The Call of the Wild* and *The Sea Wolf*. 

When did Hinkle learn German well enough to take on the daunting task of translating Jung’s magnum opus? Since it was the leading language of science in the years before World War I it is likely that she began to learn it during her medical school years and improved her command of it during her stay in Europe. Besides the translation she wrote a lengthy introduction that was released by Moffat, Yard as a separate publication [22,23]. One Greenwich Villager, Clement Wood, celebrated Hinkle in the following doggerel verse “We marched in a body to Hinkle—sort of jung sybil she were; She taught us so much about symbols and such, That we learned about women from her” ([24], p. 22). Another member of her circle was Jean Starr Untermeyer whose husband Louis did the German literary translations for *Psychology of the Unconscious*. Her memory of Hinkle had a critical edge. “But the less said about my months with Dr. Hinkle the better. I found her shallow in insight, expedient rather than constructive in her advice, and in a time of crisis—the death of my father, who incidentally, was paying for the analysis—inadequate and inhuman as well ... if, in my sessions with Dr. Hinkle, I did not progress as far in self-knowledge as I had hoped, I did ‘learn about women’ from her” ([25], p. 48).

Hinkle’s major book *The Re-Creating of the Individual* was published by Harcourt Brace in 1923. Through her membership in the Heterodoxy Club she got to know Susan Glaspell, a founding member of the Provincetown Players, and along with Eugene O’Neill, its most prolific dramatist; I would contend that *The Verge,* Glaspell’s 1921 play about a woman conducting highly unorthodox botanical experiments was partly influenced by her discussions with Hinkle about gender and creativity. Considering the theme of the play I would point out that one of Hinkle’s favorite analogies for Jung’s process of individuation was the life-work of Luther Burbank, the California “plant wizard.” She applied Burbank’s insights into plant growth in her rock garden at Roughlands, her country home in Connecticut, where she cultivated her collections of heather and barberry. 

Another of her sisters in the Heterodoxy Club was the anthropologist Elsie Clews Parsons. They would have compared the fieldwork Clews conducted among the Pueblo Indians of the Southwest with the observations Hinkle had made among the Malays in the Philippines. Parsons was a founding member of the New School for Social Research where she taught Ruth Benedict her first course in anthropology; Benedict’s interest in cultural typology later appeared in her classic study *Patterns of Culture* [26]. Hinkle divorced her husband in 1926 and later developed a close personal relationship with Katherine Thaxter. 

Hinkle attracted other women some of whom later formed what I would call the “outer circle” of the Analytical Psychology Club of New York. These included Fola LaFollette whose father, Robert, was the Progressive senator from Wisconsin; among her many activities was teaching at the City and Country School, a progressive school in Greenwich Village. Another was Margaret Doolittle Nordfeldt who had been active in the Provincetown Players and met Jung in New Mexico in 1925 and eventually became an analyst. Finally, Amy Spingarn participated in one of Hinkle’s women’s groups and went to Zurich for analysis; her husband Joel was a founding editor at Harcourt Brace, which became Jung’s American publisher. 

Another analysand was Margaret Naumburg who opened the Children’s School with a philosophy based on the work of the Zurich School of psychoanalysis and who later taught art therapy at New York University into her 80s. She had been married to Waldo Frank, a critic, who was on the editorial board of *The Seven Arts* along with James Oppenheim [27]. Aline Bernstein, a costume designer with The Neighborhood Playhouse, analyzed with Hinkle and would recount her sessions to her lover, the novelist Thomas Wolfe. Finally, Alice Lewisohn, one of the founders of the Playhouse, was attracted to Jungian psychology and spent her last years in Zurich. 

During the 1920s Hinkle’s articles about women, marriage, and education appeared frequently in such magazines as *The Nation* and *Harpers*. There are several sidebars to Hinkle’s career during that period, which are worth mentioning. She was a member of the American Society for Psychical Research and investigated Eileen Garrett, one of the most prominent mediums of the time. The other involves her friendship with an African-American celebrity figure named Lobagola. In his autobiography *Man Without a Country* (1930) he billed himself as an African who had stowed-away on a steamer to Glasgow and was finally able to make his way to America. His mix of intelligence and “primitivity” captured the imagination of the American public but it turned out that he was, in fact, not a native African but an African-American hoaxer who later dropped out of sight. We will probably never know the exact nature of their relationship, but it evokes the zaniness of the movie *Zelig* where the woman analyst Eudora Fletcher played by Mia Farrow treats the shape-shifting Trickster figure played by Woody Allen. There is a family story that Franklin Roosevelt met with Hinkle at Roughlands for help in regaining his self-confidence after contracting polio in 1921. We do know that Hinkle was acquainted with his wife Eleanor through their membership in Chi Omega, the society of professional women and that she attended at least two awards dinners at the White House. 

In 1928 Hinkle opened Smoky Hollow Lodge, a residential treatment facility in a renovated farmhouse just down the road from Roughlands. Bing Crosby spent time there for treatment of alcoholism and the *New Yorker* writer Nancy Hale, who credited Hinkle with helping her to overcome a serious case of writer’s block, wrote a fictionalized account of her stay in the novel *Heaven and Hardpan Farm* (1957). After Hinkle’s death the property was sold, one of its later owners was the artist Jim Dine who sold it to its present owner.

At this point it is important to tell something about her friendship with someone who almost became one of the founding mothers of the American Jungian movement. Her name was Constance Long and in many ways she was Hinkle’s British twin-sister. She was an M.D. and active in a number of different professional organizations and public health projects. She edited a volume of Jung’s works entitled *Collected Papers on Analytical Psychology* in 1916, the same year that *Psychology of the Unconscious* came out. In them the two women brought Jung’s new approach to psychology to the attention of the English-speaking world. In 1919 Long attended the YWCA-sponsored International Congress of Women Physicians in New York where she got acquainted with the local group. After the conference was over Kristine Mann decided to start analysis with Hinkle while Eleanor Bertine went to London to work with Long. Hinkle recalled those early years at the memorial service for Dr. Mann in 1946. “[In] about 1922, Dr. Constance Long again came to this country and in the latter part of that year Dr. Harding arrived on a brief visit to Dr. Bertine. We were now a little group of five and to celebrate that expansion we introduced our English friends over the New Year to a New England winter at my country home. We were all very congenial and our welcome to the New Year was centered around a huge open fireplace where we talked and discussed many things of mutual interest, with no intimation that one of our number would be gone in less than two months” [28]. Long suddenly took ill and died, Hinkle buried her ashes on the grounds of Roughlands, the memorial plaque is inscribed with the phrase “She followed the gleam.” She also dedicated her book *Re-Creating the Individual* (1930) to the memory of her departed friend.

What exactly was Hinkle’s relationship to Mann and Bertine? As they received their medical degrees from Cornell at a time when Hinkle was on still on staff it is possible that they knew about her before their meeting at the YWCA conference. In 1920, they all attended Jung’s Sennen Cove seminar in the U.K. and entertained him when he visited New York after his trip to the American Southwest in 1925. By this time they had been joined by Frances Wickes, a school psychologist, who found Jung’s psychological approach helpful in her practice and wrote several books from that perspective. Together they founded the Analytical Psychology Club of New York in 1936, attended the Bailey Island seminar that year, and hosted him the next after his Terry lectures at Yale. In her reminiscences Millicent Kelley remembers Emma Jung visiting Hinkle at Roughlands, which would most likely have occurred at this time.

What explains Hinkle’s being less well-known to Jungians than the others? The first thing to consider is that besides the fact she was their senior in both the professional and psychoanalytic fields she also pursued a more independent path. As an extraverted social activist she was never quite the orthodox Jungian that they were. Their more introverted orientation to the “inner journey,” evident in such works as Esther Harding’s *Women’s Mysteries*, was in contrast to her own psychologically-oriented reformism. It comes as no surprise that they opposed the use of group psycho-therapy which Hinkle pioneered, she functioned as something of a “den mother” to the young women of the Heterodoxy Club and helped organize the first of what would later be called “consciousness-raising” sessions. 

Now to her relationship with Jung. It is important to recognize that when Hinkle went to Europe she met Jung and Freud as a professional equal and not as a mere disciple. “I was compelled to follow the creative and prospective tendencies in the human being as well as the regressive and destructive ones. Although my work is closely related to Jung’s, I do not present ‘it’ [this book] as an exposition of Jung’s ideas” ([29], p. 6). She was independent enough of him to develop a psychological typology distinct from his; based on her analytic experience Hinkle divided extraverts and introverts into “Objective, Simple, and Subjective” types. She used Presidents Teddy Roosevelt and Woodrow Wilson as examples of the extraverted and introverted types and was ahead of the times in discussing bisexuality in positive terms. 

She continued to be one of Jung’s primary American contacts in the 1930s when the question of his alleged anti-Semitism became an issue. This got its most public hearing at the 1936 Harvard Tercentenary where Henry Murray came to his defense in the pages of the *Harvard Crimson*. When Jung left Boston he went directly to Bailey Island for his seminar. I think that the most likely reason for Hinkle’s going up there early was to talk to Jung about what she knew of the allegations against him and the impact they were having on his reputation in the States [30].

Hinkle remained active into her 80s when she developed bone cancer and died at Columbia-Presbyterian Hospital on February 28, 1953. Her body was cremated and the ashes scattered around the grounds of her country home; a memorial plaque was affixed to the boulder beside that of her friend Constance Long. Obituaries in the New York Times, the Herald Tribune, and a number of professional journals all recognized her pioneering role in establishing the professional status of analytic therapy in this country. The Analytical Psychology Club established the Beatrice M. Hinkle Scholarship to assist analysts wishing to study at the Jung Institute in Zurich and it operated into the 1960s. She was remembered in *Spring* (1954) this way. “Those who knew her will cherish the memory of her warm cordiality, of her love for people, of animals, of flowers, which was part of her zest for living. Here she showed the same welcoming acceptance of life in all its aspects that led her to recognize the scope and human usefulness of Dr. Jung’s theories. Dr. Hinkle has left us a lasting example of a full, productive, and courageous life.” 

What is Beatrice Hinkle’s legacy? She agreed with Jung’s critique of Freud’s theoretical premises and concurred with his new focus on the importance of the mother and the role of creative fantasy in overcoming current life problems. “[Jung’s] real interest lies in the forward striving principle or progressive element of life” ([29], p. 26). She taught Jung’s approach to the psyche to a network of young Greenwich Villagers who expressed their creativity in such fields as art, costume and set design, and progressive education. Finally, her feminist critique of the patriarchal bias of psychoanalysis came years before that of better-known writers from the Women’s Movement of the 1960s. Hinkle was a pioneering American woman analyst whose contributions to the development of Jungian psychology in the United States deserve greater recognition today.
